# Post-operative intracranial gas migration with optic nerve infiltration and atrophy following retinal detachment repair

**DOI:** 10.1016/j.ajoc.2020.100920

**Published:** 2020-09-06

**Authors:** James M. Harris, Ian C. Han, Mira M. Sachdeva, Alice Y. Zhang, Nazlee Zebardast

**Affiliations:** a Harvard-MIT Division of Health Sciences and Technology, Harvard Medical School, 260 Longwood Ave, Boston, MA, 02115, USA; b Institute for Vision Research, Department of Ophthalmology and Visual Sciences, Carver College of Medicine, University of Iowa, 200 Hawkins Drive, Iowa City, IA, 52242, USA; c Wilmer Eye Institute, Johns Hopkins University, 600 N. Wolfe Street, Baltimore, MD, 21287, USA; d Department of Ophthalmology, The University of North Carolina at Chapel Hill, 2226 Nelson Highway #200, Chapel Hill, NC, 27517, USA; e Department of Ophthalmology, Massachusetts Eye and Ear Infirmary and Harvard Medical School, 243 Charles Street, Boston, MA, 02114, USA

**Keywords:** Glaucoma, Optic nerve, Pneumocephalus, Retina, Retinal detachment, Vitrectomy

## Abstract

**Purpose:**

To report a patient with post-operative gas migration into the optic nerve and lateral ventricles after retinal detachment repair.

**Observations:**

A 78-year-old pseudophakic man developed a temporal visual field cut in his non-operative, right eye 3 weeks after repair of a recurrent, shallow, macula-involving retinal detachment with perfluoropropane intraocular gas in the left eye. Visual acuity in the right eye measured 20/40, and static perimetry demonstrated temporal visual field loss that respected the vertical midline. Dilated fundus examination of the right eye was unrevealing for any retinal cause, raising suspicion for an intracranial etiology. An urgent CT scan of the brain demonstrated gas in all segments of the left optic nerve and lateral ventricles, consistent with intracranial gas migration along the optic nerve. Given the absence of systemic neurologic symptoms, cautious observation was advised on consultation with neuroradiology and neurosurgery, and follow-up CT scan 1 week later showed resolution of the intracranial gas. By 10-weeks post-operatively, vision returned to 20/20 in the right eye with persistent temporal field loss, and the left eye was hand motions (20/70 pre-operatively) with evidence of optic nerve atrophy and severe cupping.

**Conclusions:**

Intracranial gas migration is a rare complication of retinaldetachment repair with intraocular gas and may occur in the setting of structural defects of the optic nerve and high post-operative intraocular pressure. Clinicians should be alert to this rare but serious complication, which can cause neurologic symptoms and result in vision loss in both the operative and non-operative eyes.

## 1 Introduction

Rhegmatogenous retinal detachments are vision-threatening pathology frequently encountered by vitreoretinal surgeons. To repair these detachments, material such as gas or silicone oil is often injected into the vitreous chamber following vitrectomy to help reapproximate and tamponade the retina to the underlying retinal pigmented epithelium. Complications of intraocular gas use, while rare, can result in significant morbidity including ocular hypertension, hypotony, and migration of gas to the anterior chamber where it can disrupt the corneal endothelium, the subretinal space, or into the optic nerve and even the brain parenchyma, where it can cause neurologic symptoms and vision loss.^1^, ^2^, ^3^ Deaths have even occurred from presumed venous air embolism after fluid-air exchange.^4^ Here we report a case of the rare complication of gas migration into the brain with associated visual field loss in the fellow eye and resultant poor vision in the operative eye following repair of a retinal detachment with pars plana vitrectomy.

## 2 Case report

A 73-year-old pseudophakic male presented for evaluation of a recurrent, inferotemporal, macula-involving retinal detachment in his left eye. He had a history of two prior retinal detachments complicated by proliferative vitreoretinopathy in this eye, initially repaired with pars plana vitrectomy (PPV), endolaser, and sulfur hexafluoride (SF6) gas placement, followed six months later with repeat PPV, scleral buckle, membrane peel, drainage retinotomy, and perfluoropropane (C3F8) gas tamponade.

Fourteen months after his most recent retinal detachment repair, the patient presented with a new shadow in the inferior visual field of his left eye. On examination, visual acuity with correction measured 20/20 in the right eye and 20/70 with improvement to 20/30 with pinhole in the symptomatic left eye. Intraocular pressure (IOP) measured 12 mmHg and 9 mmHg in the right and left eye, respectively. The optic nerves were noted to have borderline enlarged vertical cup-to-disc ratio (vCDR) of 0.6 bilaterally. Dilated fundus examination of the left eye demonstrated a recurrent inferotemporal retinal detachment with shallow fluid in the inferotemporal macula, and contraction of the retina off of prior laser inferotemporally due to grade C proliferative vitreoretinopathy.

He underwent uncomplicated repair with PPV, subretinal fluid drainage with assistance of perfluoro-*n*-octane (PFO), endolaser, and 14% C3F8 gas tamponade, followed by typical face-down post-operative positioning. The case was done under monitored anesthesia care with retrobulbar block (2% lidocaine and 0.75% bupivacaine) after induction with propofol, and no inhaled anesthetic agent was used that may have altered the partial pressure of the intraocular gas. No optic nerve changes were appreciated during the case, including during placement and removal of PFO, or air-fluid exchange. The patient was hemodynamically stable throughout the course of the procedure, with no episodes of hypotension. The optic nerve and retinal vasculature were observed to be well-perfused on post-operative examination. On post-operative day 1, vision measured count fingers, IOP was 18 mmHg, and the retina was attached under a 90% gas fill. The examination remained stable at post-operative week (POW1), except for an IOP of 26 mmHg with 80% remaining gas fill, at which time he was started on topical dorzolamide/timolol.

**Fig. 1:**
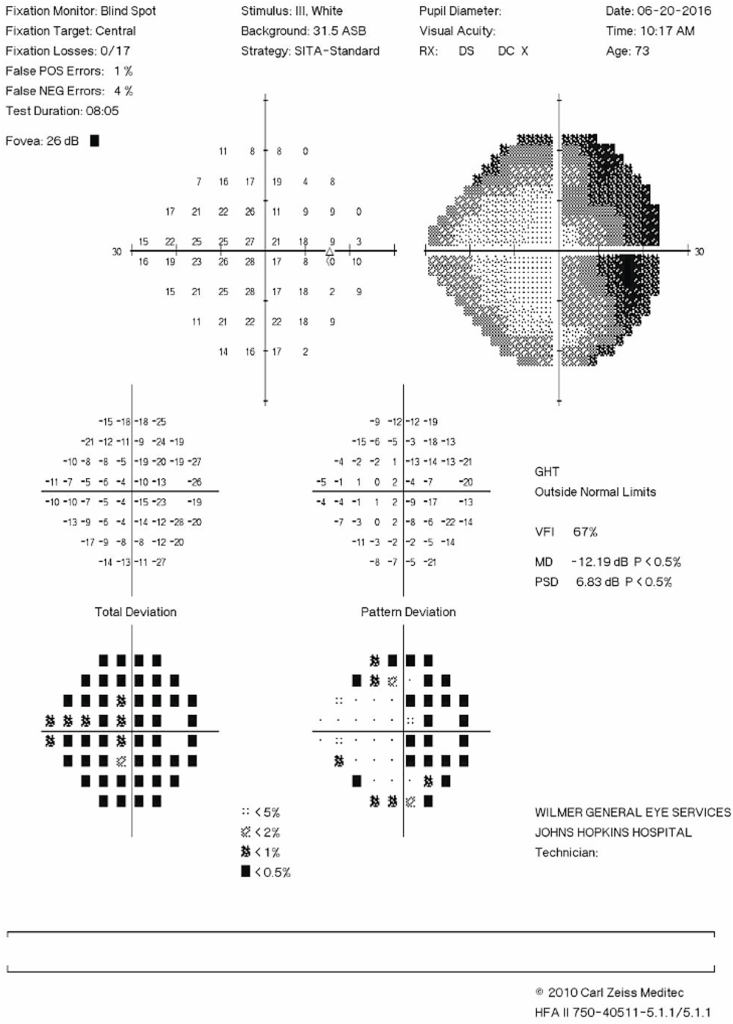
**Visual field defect in nonoperative eye.** Humphrey SITA standard 24–2 visual field test obtained 3 weeks post-operatively shows temporal field defects of the right eye respecting the vertical meridian.

Three weeks post-operatively, he presented urgently for new darkening of temporal vision in the nonoperative right eye in the absence of flashes, floaters or pain. He had no numbness, weakness, or other neurologic symptoms. Visual acuity measured 20/60 in the right eye and light perception in the left eye. No definite afferent pupillary defect was noted initially, but assessment was challenging due to the post-operative dilation and presence of 40% gas tamponade in the left eye. However, at follow-up on POW6 an afferent pupillary defect by reverse was observed. Static perimetry confirmed temporal visual field loss respecting the vertical meridian in the right eye (Fig. 1) and dilated fundus examination of the right eye demonstrated no visible retinal pathology, raising suspicion for an intracranial etiology.

**Fig. 2:**
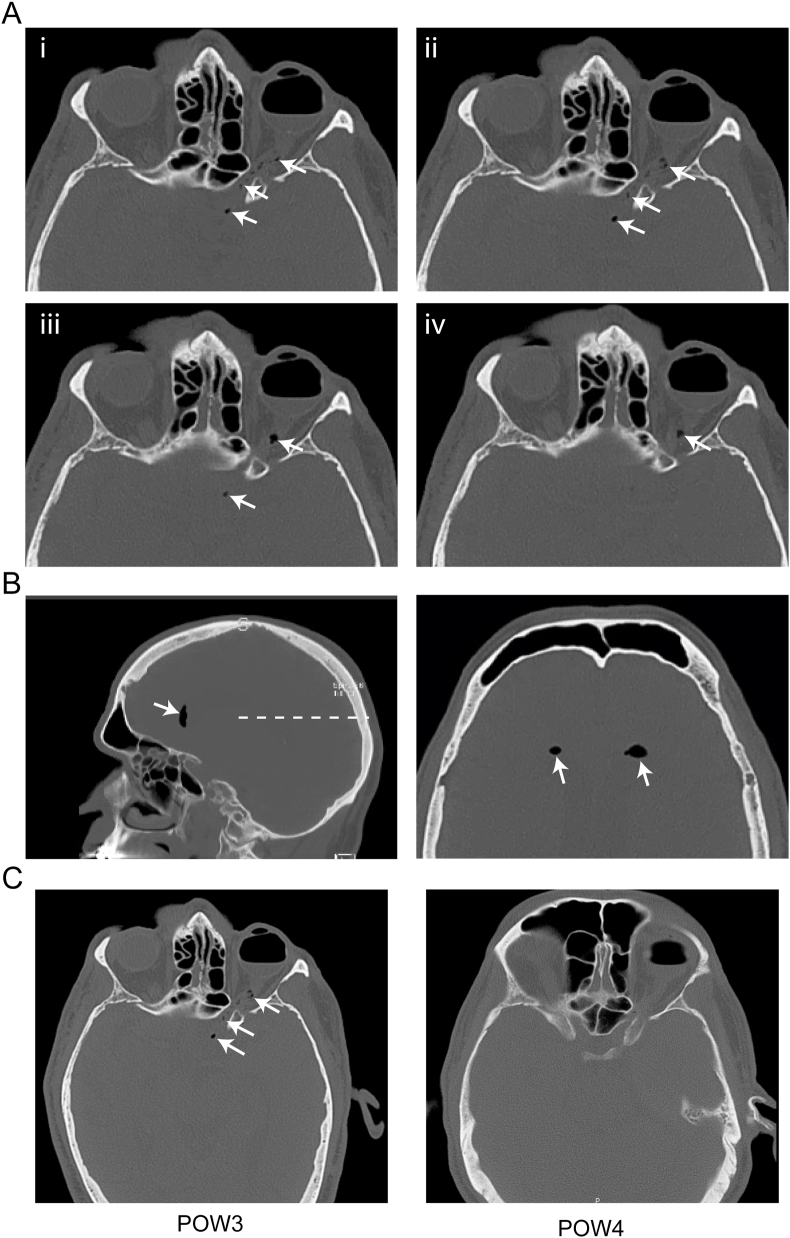
**Intracranial gas migration and subsequent resolution. (A)** Inferior-to-superior axial series from a non-contrast CT scan of the head at post-operative week 3 shows about a 60% gas fill in the vitreous cavity of the left eye (i-iv). Radiolucent spaces are seen infiltrating the intraorbital, canalicular, and prechiasmatic segments of the optic nerve (white arrows), consistent with intracranial extension of gas. **(B)** Sagittal view of the same CT scan in (A) shows pneumocephalus in the anterior horn of the lateral ventricle (left, white arrows). An axial view at the level of the dashed line in the left image demonstrates bilateral pneumocephalus (right, white arrows). **(C)** An axial image from the same CT scan at post-operative week 3 (POW3) as in (A) shows radiolucent spaces in the optic nerve (left, white arrows). Follow up non-contrast axial CT scan one week later (POW4) shows about a 50% intraocular gas fill with resolution of the previously-observed radiolucent spaces along the optic nerve and within the ventricles (right). These CT images were taken from a similar plane at the level of the optic nerve, however other cranial structures are out-of-plane due to differences in head positioning.

The patient was referred urgently to the emergency department for evaluation, where a non-contrast axial CT scan of the brain was obtained, revealing intraocular gas fill, as well as foci of gas in the intraorbital and canalicular segments of the left optic nerve, extending to the optic chiasm (Fig. 2A, arrows), suspicious for intracranial extension of the intraocular gas tamponade. There was also pneumocephalus in the frontal horns of the lateral ventricles (Fig. 2B, arrows). No other intracranial abnormalities or signs of acute infarct were noted. Neuroradiology and neurosurgery were consulted, and given the otherwise nonfocal neurologic examination, close, cautious observation was advised with follow up CT scan in one week, which showed resolution of the pneumocephalus and optic nerve gas (Fig. 2C).

**Fig. 3:**
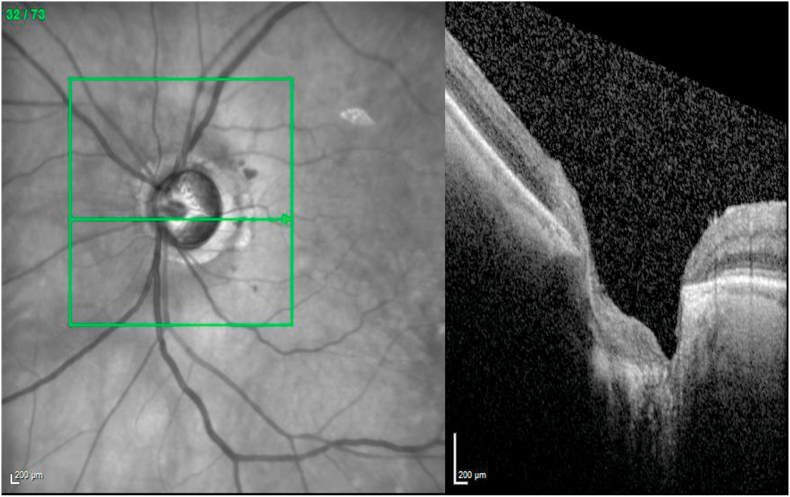
**Acute optic nerve degeneration in operative eye.** Optical coherence tomography of the left optic nerve at post-operative week 10, after resolution of pneumocephalus, showed severe excavation, but no distinct optic pit. The cup-to-disc ratio was 0.9 with marked superior and inferior thinning.

One month later (POW10), vision had returned to 20/20 in the right, but the temporal field defect persisted and the left eye remained light perception only. Dilated fundus examination showed a healthy appearing optic nerve with vCDR 0.65 and no pit or structural changes in the right eye, and diffuse optic nerve pallor with enlargement of vCDR to 0.9 and corresponding superior and inferior optic nerve rim thinning in the left eye. Dedicated optical coherence tomography (OCT) volume scan of the optic nerve demonstrated significant optic disc excavation and loss of retinal nerve fiber layer, but no definitively visible break in the lamina cribosa (Fig. 3). The vitreous was noted to be optically empty, with complete dissolution of gas within the vitreous cavity, and the retina was attached without proliferative vitreoretinopathy. At his most recent follow up in September of 2019, three years after initial presentation, his vision remained 20/20 on the right with persistent temporal defect and no light perception on the left.

## 3 Discussion

Migration of exogenous intraocular material into the optic nerve and lateral ventricles is an exceedingly rare, but serious complication of retinal detachment repair. This complication has most frequently been reported after use of intraocular silicone oil,^5^, ^6^, ^7^, ^8^, ^9^, ^10^ possibly due to its permanent nature or differences in physical properties, such as lower surface tension, though there has been at least two other cases of intracranial gas migration.^2^^,^^3^ In one instance, accidental injection of a high concentration of expansile gas led to intraocular pressures reaching 60 mmHg in an eye with known previous glaucomatous optic nerve atrophy.^3^ The second case of intracranial gas migration similarly involved elevated IOP reaching 88 mmHg, due to use of nitric oxide anesthesia for an urgent procedure, which can quickly diffuse into the gas filled eye and subsequently expand.^2^ Once in the brain parenchyma, gas may cause a variety of neurologic symptoms including headache, focal weakness, and vision loss, which unfortunately may be permanent.^2^^,^^3^^,^^5^

A variety of mechanisms have been proposed to explain how intraocular material can migrate all the way to the lateral ventricles, including the presence of traumatic^9^ or congenital^11^ defects at the optic nerve head, and IOP-based etiologies.^12^ In previous reports of intracranial gas migration, it was speculated that a possible, though unobserved, congenital structural defect of the optic nerve, such as an optic pit or coloboma, may have enabled intracranial gas escape.^3^ These congenital defects are the result of failed closure of the optic fissure, potentially allowing communication between the vitreous cavity and subarachnoid space.^11^^,^^13^ Once in the subarachnoid space, gas or oil could retrogradely enter the ventricular system through the foramina of Luschka and Magendie in the posterior cranial fossa. Indeed, intracranial migration of silicone oil has been observed in patients with these congenital structural defects.^11^^,^^13^ Theoretic models have calculated that a pressure gradient of roughly 11 mmHg, within the range of physiologic variation, could allow passage of intraocular gas into subretinal and subarachnoid compartments through structural defects.^13^ However, since both prior cases of intracranial gas migration involved markedly elevated IOPs and no congenital defects were observed, an etiology involving pathologic ocular hypertension is also possible.

In particular, rather than traveling through a preexisting channel, it has been hypothesized that increased intraocular pressure can drive intraocular material into an atrophic optic nerve, across the pia mater, and into the subarachnoid space, where it could then enter the ventricular system through the foramina in the posterior cranial fossa.^12^ In addition to this relatively circuitous route, we speculate that a more direct path of migration may also contribute in some patients. It is interesting that the path of gas migration from the eye through the optic nerve, to the chiasm, and ultimately into the ventricular system is highly reminiscent of a fluid connection between these structures that exists transiently during embryonic development. Specifically, the eye is formed from outpouchings of the neural tube, which contact the surface ectoderm to induce globe morphogenesis.^14^ The primordial optic nerve, the optic stalk, contains a fluid filled lumen that is continuous with the third ventricle.^15^ Retinal ganglion cell axons projecting from the retina to the thalamus eventually fill in this optic stalk lumen, obliterating it to form the optic nerve.^15^ We speculate that acute atrophy and degeneration of these retinal ganglion cell axons may enable partial re-opening of this developmental connection, allowing intraocular material to travel within the parenchyma of the optic nerve, ultimately entering the ventricular system directly where the optic chiasm contacts the 3rd ventricle.

In support of this mechanism, histopathologic analysis of an eye enucleated due to severe glaucoma following silicone oil implantation revealed globules of oil in the parenchyma of the optic nerve, extending fully to the line of surgical transection with coalesced foci forming cavernous spaces along the way.^16^ This pattern closely resembles the pockets of gas in the optic nerve observed on CT scan in patients with intracranial gas migration. Experimental models of acute glaucoma offer further support. Specifically, induction of acute glaucoma in owl monkeys with IOPs ranging from 40 to 80 mmHg led to necrotic degeneration of the optic nerve with vitreous fluid filling large cavernous spaces throughout the parenchyma of the injured nerve.^17^ IOPs in this range were observed in the previously reported cases of intracranial gas migration in patients, consistent with a mechanism of intraparenchymal migration through pathways opened after acute glaucomatous optic nerve degeneration from pathologic intraocular pressure elevation.^2^^,^^3^

In contrast to the other cases of intracranial gas migration that resulted after marked ocular hypertension from overfilling of the vitreous cavity with gas, in this case, migration occurred despite moderately elevated pressures and no error in the amount of gas filling. Although IOP was only moderately elevated, acute axonal degeneration likely occurred, as evidenced by optic nerve pallor, severe excavation, notching, and increased vCDR from 0.6 to 0.9 by POW10. However, the initial cause of acute axonal atrophy is less clear. Perhaps this patient's optic nerve was extremely sensitive to even moderate ocular hypertension, IOPs in the post-operative period reached higher levels that what was measured at clinic visits, or the gas itself was toxic and induced atrophy in this patient, which could potentially explain the hemianopia from atrophy of crossing fibers exposed to gas at the chiasm. Regardless of the cause of nerve atrophy, a similar mechanism as proposed above may apply here, in which acute retinal ganglion cell axonal degeneration cleared a path for intraocular gas to travel from the vitreous chamber directly to the 3rd ventricle. It is also possible that the gas traveled the an alternate, more circuitous route, possibly through the subarachnoid space to the ventricular system, though no congenital structural defect of the optic nerve was observed.

Curiously, intraocular gas is routinely used in patients with underlying glaucoma and optic pits, and yet reports of intracranial gas migration are extremely rare. Perhaps gas migration is more prevalent, but asymptomatic and transient, so migration does not present clinically. Additionally, in patients with chronic glaucomatous optic nerve degeneration, space created by axonal loss may be soon filled in with fibrosis or astrogliosis.^18^ Thus, gas migration may only be possible in the context of rapid cavernous nerve degeneration in a narrow window following intraocular gas instillation, but before fibrosis or gas resorption. Overall, this complication, while rare, suggests the importance of careful ocular pressure monitoring and management following placement of intraocular silicone oil or gas, particularly in patients susceptible to optic nerve atrophy or with underlying congenital defects.

## 4 Conclusions

Repair of retinal detachments with intraocular gas can lead to a rare complication of intracranial gas migration through a structurally aberrant optic nerve. This gas migration can cause neurologic symptoms, as well as permanent visual loss in both operative and non-operative eyes.

## Patient consent

Consent to publish the case report was not obtained. This report does not contain any personal information that could lead to the identification of the patient.

## Funding

J.M.H acknowledges support from the 10.13039/100000057National Institute of General Medical Sciences (T32GM007753).

## Authorship

All authors attest that they meet the current ICMJE criteria for Authorship.

## Declaration of competing interest

The following authors have no financial disclosures: JMH, ICH, MMS, AYZ, NZ.
